# Establishment of primary cell culture and an intracranial xenograft model of pediatric ependymoma: a prospect for therapy development and understanding of tumor biology

**DOI:** 10.18632/oncotarget.24932

**Published:** 2018-04-24

**Authors:** Lorena Favaro Pavon, Tatiana Tais Sibov, Silvia Regina Caminada de Toledo, Daniela Mara de Oliveira, Francisco Romero Cabral, Jean Gabriel de Souza, Pamela Boufleur, Luciana C. Marti, Jackeline Moraes Malheiros, Edgar Ferreira da Cruz, Fernando F. Paiva, Suzana M.F. Malheiros, Manoel A. de Paiva Neto, Alberto Tannús, Sérgio Mascarenhas de Oliveira, Nasjla Saba Silva, Andrea Maria Cappellano, Antonio Sérgio Petrilli, Ana Marisa Chudzinski-Tavassi, Sérgio Cavalheiro

**Affiliations:** ^1^ Department of Neurology and Neurosurgery, Escola Paulista de Medicina (EPM), Universidade Federal de São Paulo (UNIFESP), São Paulo, Brazil; ^2^ Pediatric Oncology Institute, Grupo de Apoio ao Adolescente e à Criança com Câncer (GRAACC), Escola Paulista de Medicina (EPM), Universidade Federal de São Paulo (UNIFESP), São Paulo, Brazil; ^3^ Department of Genetics and Morphology, Universidade de Brasília, Brasília, Brazil; ^4^ Hospital Israelita Albert Einstein (HIAE), São Paulo, Brazil; ^5^ Biochemistry and Biophysics Laboratory, Butantan Institute, São Paulo, Brazil; ^6^ Allergy and Immunopathology Graduate Program, Faculdade de Medicina, Universidade de São Paulo (USP), São Paulo, Brazil; ^7^ Department of Physiology, Universidade Federal de São Paulo (UNIFESP), São Paulo, Brazil; ^8^ Discipline of Nephrology, Escola Paulista de Medicina (EPM), Universidade Federal de São Paulo (UNIFESP), São Paulo, Brazil; ^9^ São Carlos Institute of Physics, Universidade de São Paulo (USP), São Paulo, Brazil

**Keywords:** primary culture EPN cells, pluripotency markers, animal model, MRI, preclinical studies

## Abstract

**Background:**

Ependymoma (EPN), the third most common pediatric brain tumor, is a central nervous system (CNS) malignancy originating from the walls of the ventricular system. Surgical resection followed by radiation therapy has been the primary treatment for most pediatric intracranial EPNs. Despite numerous studies into the prognostic value of histological classification, the extent of surgical resection and adjuvant radiotherapy, there have been relatively few studies into the molecular and cellular biology of EPNs.

**Results:**

We elucidated the ultrastructure of the cultured EPN cells and characterized their profile of immunophenotypic pluripotency markers (CD133, CD90, SSEA-3, CXCR4). We established an experimental EPN model by the intracerebroventricular infusion of EPN cells labeled with multimodal iron oxide nanoparticles (MION), thereby generating a tumor and providing a clinically relevant animal model. MRI analysis was shown to be a valuable tool when combined with effective MION labeling techniques to accompany EPN growth.

**Conclusions:**

We demonstrated that GFAP/CD133+CD90+/CD44+ EPN cells maintained key histopathological and growth characteristics of the original patient tumor. The characterization of EPN cells and the experimental model could facilitate biological studies and preclinical drug screening for pediatric EPNs.

**Methods:**

In this work, we established notoriously challenging primary cell culture of anaplastic EPNs (WHO grade III) localized in the posterior fossa (PF), using EPNs obtained from 1 to 10-year-old patients (*n* = 07), and then characterized their immunophenotype and ultrastructure to finally develop a xenograft model.

## INTRODUCTION

Ependymal tumors are neuroepithelial malignancies of the central nervous system (CNS) that occur in both children and adults [1]. These tumors can develop along the entire neuroaxis, comprising the hemispheres, the hindbrain, and the spinal cord [2]. In children, 90% of ependymomas (EPNs) occur intracranially; two-thirds of those EPNs are located in the posterior fossa and one-third within the supratentorial compartment [3]. Considering all age groups, more than 20% of primary spinal cord tumors are of ependymal lineage [4]. The clinical behavior of ependymal tumors is highly variable, and approximately 40% of cases are incurable because of the paucity of the effective treatment options available [4, 5]_._ The 10-year overall survival rate is approximately 64% in pediatric patients and ranges from 70% to 89% in adult patients [4].

Despite recent improvements in neurosurgery, neuroimaging techniques and post-operative adjuvant therapy, the prognosis for pediatric EPNs continues relatively poor when compared with other pediatric brain tumors. The 5-year survival rate spans from 39% to 64%, with a 5-year progression-free survival rate of 23% to 45% [6]. In addition, late relapses up to 15 years after initial treatment are not uncommon [7]. The ability to predict patient outcome has been hampered by the heterogeneous clinical behavior of EPNs in children, insufficient recruitment into large prospective clinical trials, and contradictory studies of existing clinicopathological prognostic markers [8, 9]. EPNs are classified according to their anatomical location in nervous system (posterior fossa, supratentorial and spine); and more recently, also based on DNA methylation profiling [10].

Poor outcomes and the unpredictable behavior of this tumor in children have shifted attention to improving our knowledge of EPN biology [3]. Consequent advances have been made, including the identification of biological prognostic markers for children with intracranial EPN. The limited availability of *in vitro* and *in vivo* model systems has hampered efforts to understand EPN tumor ultramorphology, immunophenotypic markers of pluripotency in primary culture and tumor behavior. We addressed this lack by developing experimental models for EPNs that replicated the histopathological phenotypes of the parent EPN.

Yu and coworkers [11] successfully developed a xenograft model of EPN by transplanting a fresh surgical EPN tissue from a pediatric patient into the brain of immune deficient mice. Further, a permanent cell line (BXD-142EPN) was derived from a passage II of the xenograft tumor [11]. Using the same strategy, deriving cell lines by human xenograft tissue specimens, Guan *et al.* [12] established two EPN cell lines. Johnson and coworkers [13] developed a mouse model by selecting neuronal stem cells with a deleted *Ink4a/Arf* locus that overexpress tyrosine receptor ephrin (EphB2). The same group have employed this mouse EPN model in a multi-platform drug approaches to identify selective toxicity against ependymoma cells [14]. However, a straightforward protocol to derive patient-primary EPN cells would be very useful, especially if this cells could be further generate an EPN experimental model. Here, we aimed to establish EPN primary cell isolation, culture protocol and an EPN rat experimental model using these primary cells.

**Figure 1:**
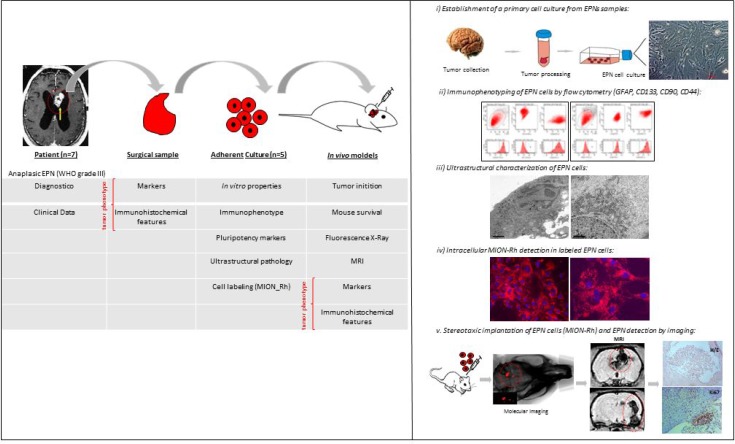
Illustration of experimental hypothesis demonstrated in 5-step model (**i**) establishment of a primary culture of anaplastic EPN cells (WHO grade III), from the PF of 1–10-year-old patients to the fourth cell passage; (**ii**) ultrastructural characterization of EPNs; (**iii**) evaluation of the expression levels of GFAP (tumor glial marker), CD133 (tumor neural stem cell marker), Nestin (immature neural stem cell marker), SSEA-3 (stage-specific embryonic antigen 3), CD44 (a cell-surface glycoprotein involved in cell-cell interactions), CD90 (stem/progenitor cell marker) and CXCR4 (CXC receptor 4, involved in tumor development and cells migration); (**iv**) *in vitro* labeling of a primary culture of EPN cells with multimodal iron oxide nanoparticles (MION) conjugated to Rhodamine-B (Rh-B) MION-Rh; and v) establishment of an experimental model by intracerebroventricular infusion of EPN cells and subsequent tumor monitoring by MION-Rh detection using T2- and T2^*^-weighted MRI at a field strength of 2T.

Considering the aforementioned limitations, the objective of the present study was to establish and characterize a primary culture of human EPN cells with the aim of advancing to a future experimental EPN model. We established the following 5-step model (illustrative Figure 1): (i) establishment of a primary culture of anaplastic EPN cells (WHO grade III), located in the posterior fossa (PF), from the PF of 1–10-year-old patients to the fourth cell passage; (ii) ultrastructural characterization of EPNs; (iii) evaluation of the expression levels of GFAP (tumor glial marker), CD133 (tumor neural stem cell marker), Nestin (immature neural stem cell marker), SSEA-3 (stage-specific embryonic antigen 3), CD44 (a cell-surface glycoprotein involved in cell-cell interactions), CD90 (stem/progenitor cell marker) and CXCR4 (CXC receptor 4, involved in tumor development and cells migration); iv) *in vitro* labeling of a primary culture of EPN cells with multimodal iron oxide nanoparticles (MION) conjugated to Rhodamine-B (Rh-B) MION-Rh; and v) establishment of an experimental model by intracerebroventricular infusion of EPN cells and subsequent tumor monitoring by MION-Rh detection using T2- and T2^*^-weighted MRI at a field strength of 2T.

## RESULTS

### Establishment of a primary cell culture from EPN samples

**Figure 2:**
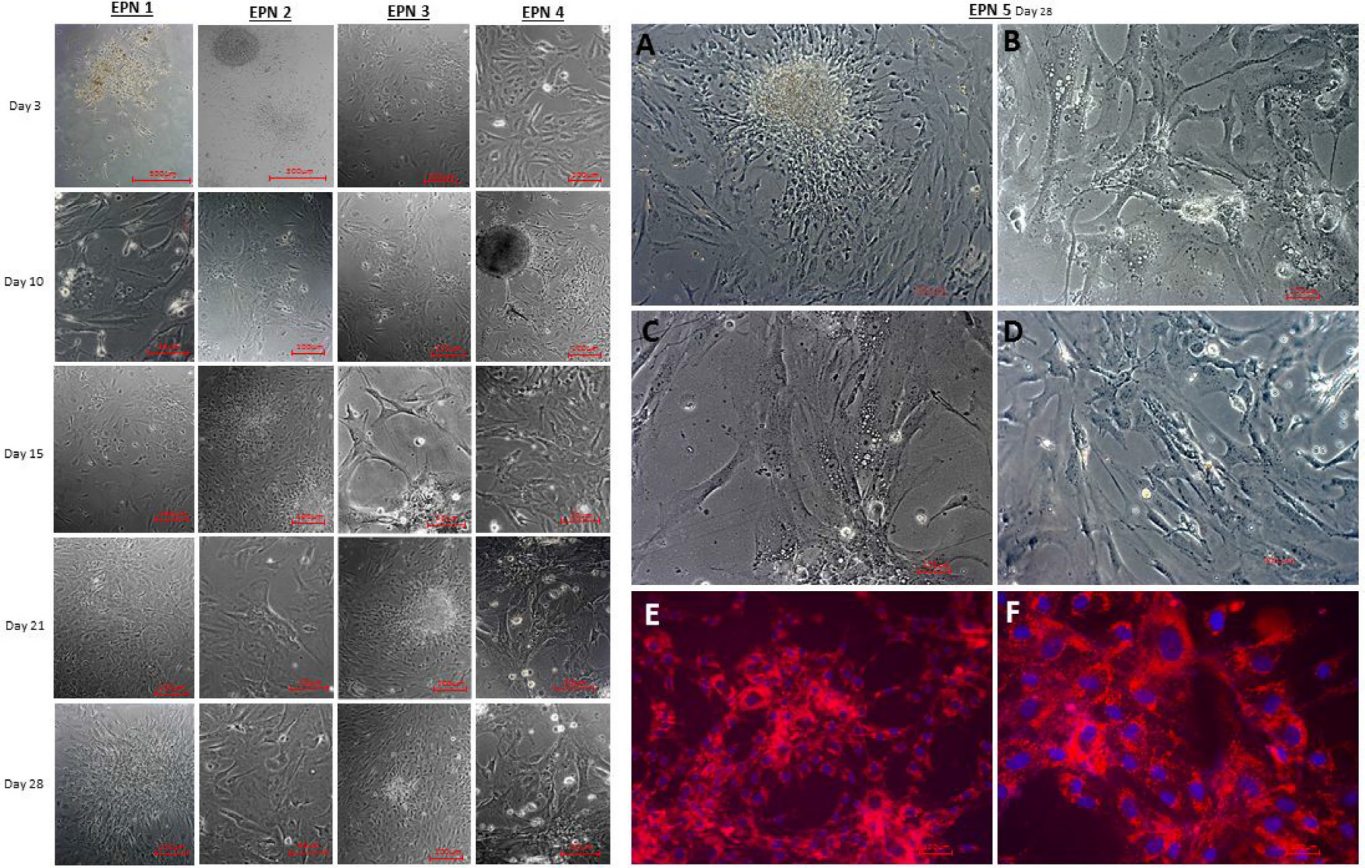
Establishment of a primary cell culture of human five EPN samples, demonstrating the profile of cell proliferation from 3 to 28 days (**A**–**D**) Establishment of a primary cell culture of human EPN_5 at the fourth cell passage. (**E**, **F**) Detection of MION-Rh labeled EPN_5 cells by fluorescence assay. These images are representative of all EPN samples. Scale bar: 100 μm.

Primary cell cultures were successfully obtained from five EPN tumor samples. The success rate of isolating EPN cell cultures from all samples was around 70%. After plating, the resulting cells were homogenous, displayed a fusiform format and were arranged in multidirectional bundles in culture (Figure 2A–2D). Figure 2 shows the profile of proliferation cell of five EPN samples from the third to the twenty-eighth day. We used all five established cellular lineages (EPN 1-5) for the experiments described within this study.

### Immunophenotyping profile of EPN-initiating cells

**Figure 3:**
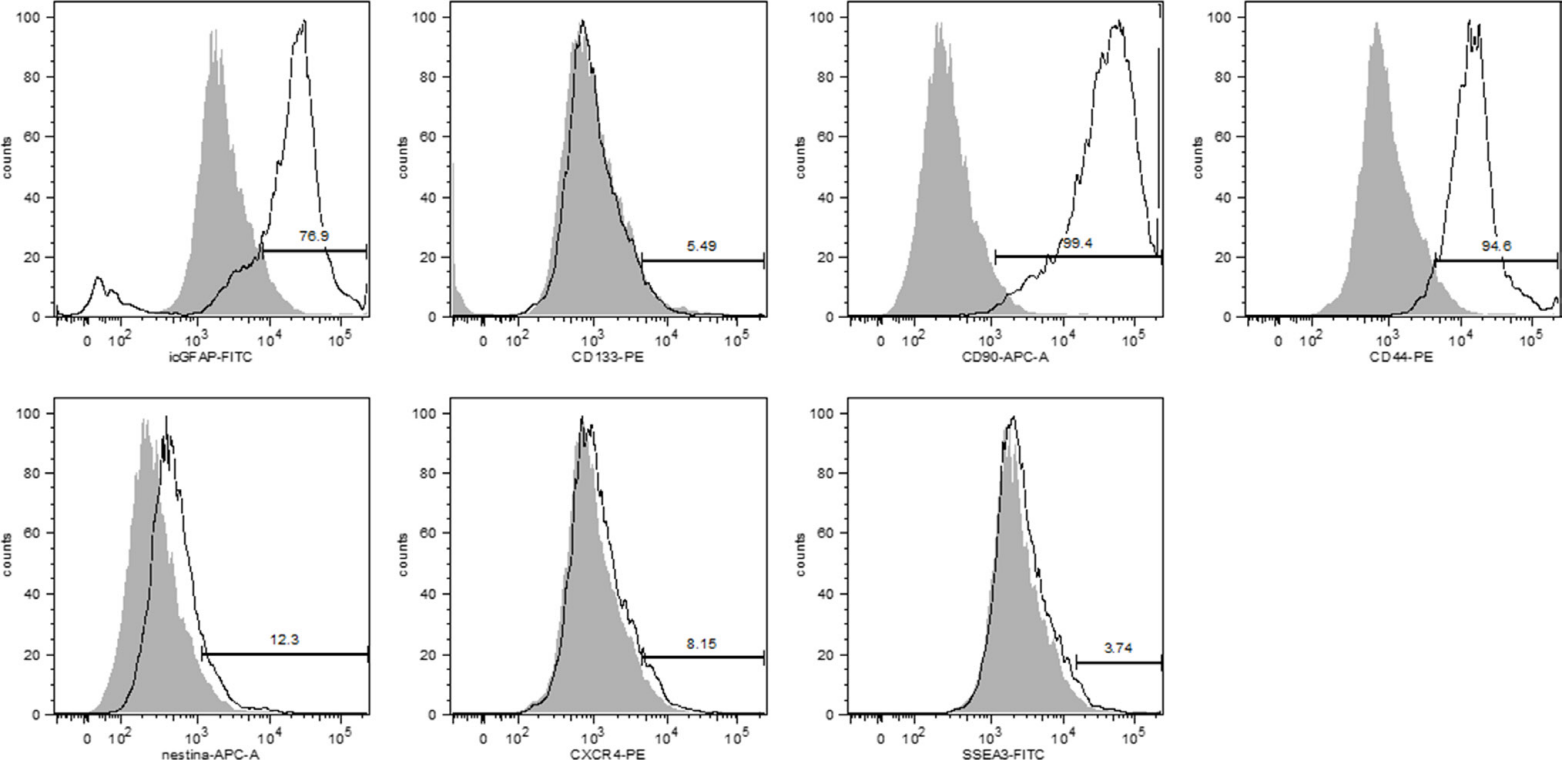
Immunophenotypic analysis showed that primary cell cultures of human EPNs expressed GFAP (76.0%), CD90 (99.4%) and CD44 (94.6%) at high rates but CD133 (5.5%), nestin (12.3%), CXCR4 (8.2%) and SSEA-3 (3.7%) at relatively low rates

Flow cytometric analyses showed that 76.9% of EPN cells expressed GFAP, a tumor glial marker, while 5.5% showed positive staining for CD133, and an important percentage (99.4%) of these cells also expressed CD90 (Figure 3). Low percentages of EPN cells also expressed Nestin (12.3%) and CXCR4 (8.15%), and only 3.74% of the cells showed positive staining for SSEA-3 (Figure 3). In addition, the majority (94.6%) of the EPN cells expressed CD44.

### Ultrastructural characterization of EPN cells

**Figure 4:**
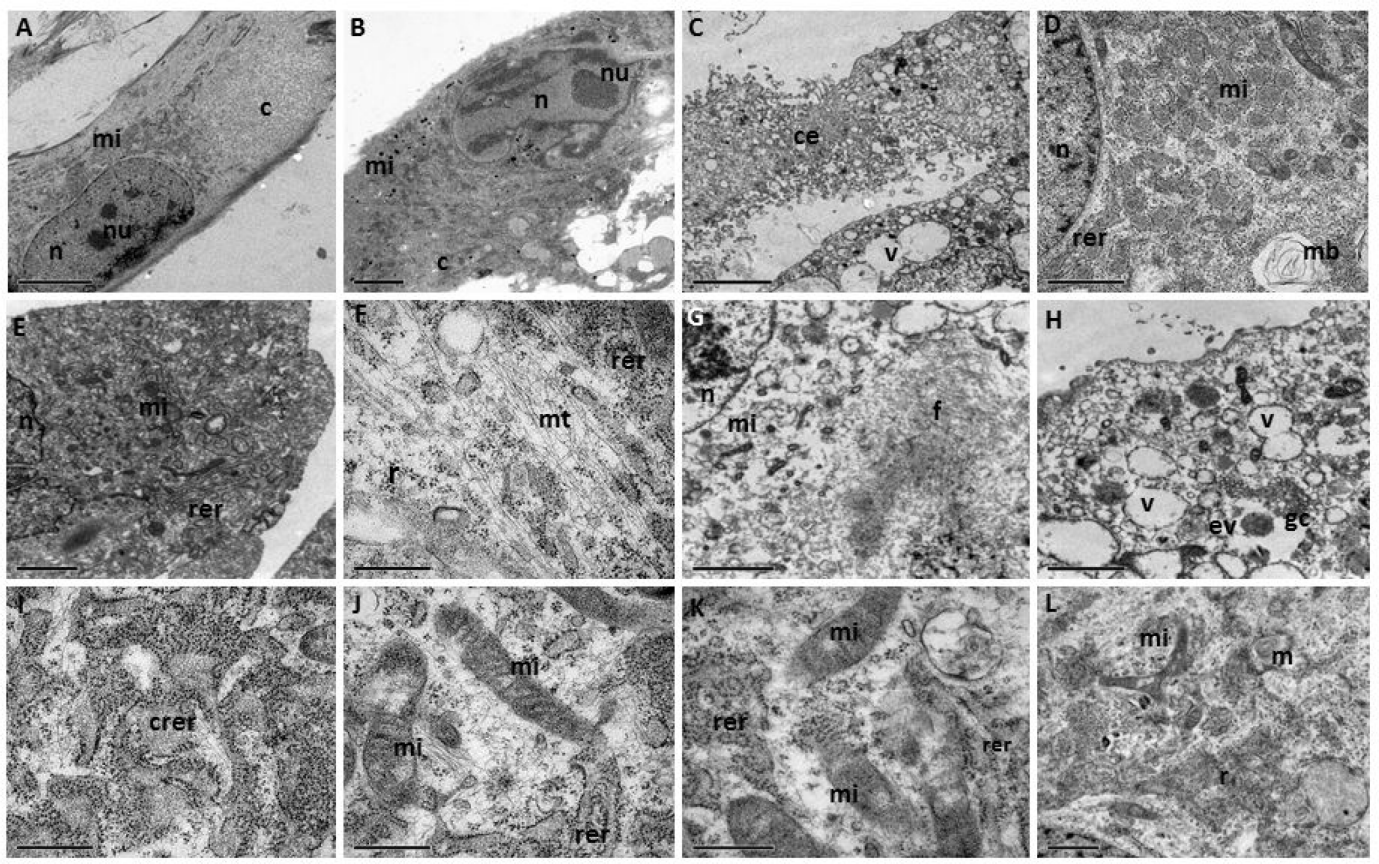
**(A**–**L**) TEM of a primary cell culture of human EPNs. n = nucleus; c = cytoplasm, nu = nucleoli; mi = mitochondria; rer = rough endoplasmic reticulum; crer = cisternae of rough endoplasmic reticulum; mt = microtubules; gc = Golgi complex; v = vacuoles; f = intermediate filament; mb = myelinic bodies; ce = cytoplasmic extensions; r = ribosomes; ev = electron-lucid vesicles. Scale bars: (A, B, E) 5.0 μm; (C, D, F-K) 2.0 μm; (L) 1.0 μm.

Ultrastructural analysis of the EPN cells supported the cytological description, i.e., spindle morphology with cytoplasmic extensions (Figure 4A–4C) and oval nuclei (Figure 2A, 2B). The cell nuclei contain heterochromatin bundles and nucleoli (Figure 4A, 4B).

The main cytoplasmic characteristic exhibited by EPN cells is the presence of numerous mitochondria with evident crests, with varying morphologies, from small and electron-dense (Figure 4B, 4D, 4E, 4G, 4L) to large and elongated (Figure 4J, 4K).

A notable number of free ribosomes (many of which were rosettes) (Figure 4F, 4L), together with rough endoplasmic reticulum (Figure 4D, 4E, 4F, 4K, 4L), were evidenced in cisternae (Figure 4I) and also observed in the cytoplasm. Golgi apparatus with slightly dilated cisterns and vacuoles were observed (Figure 4H). Electron-lucid vesicles (Figure 4H), microtubules (Figure 4F) and myelinic bodies (Figure 4D, 4L) were also present. The cytoplasm contained bundant intermediate filaments (Figure 4G).

### Detection of MION-Rh labeled EPN cells using a fluorescence assay

For visualization in tumorigenicity tests, EPN cells were labeled with multimodal iron oxide nanoparticles conjugated with Rhodamine-B (MION-Rh). Rhodamine-B is a fluorescent dye that can be visualized by both MRI and fluorescence imaging. The intracellular distribution of MION-Rh in EPN cells was qualitatively evaluated using fluorescence microscopy with an Rh-B filter (530 nm and 550 nm). We observed that MION-Rh nanoparticles were internalized by EPN cells and formed intracellular granules or small fluorescent red clusters (Figure 2E, 2F).

### Formation of orthotopic xenograft EPN: *In vivo* tumor detection by imaging

**Figure 5:**
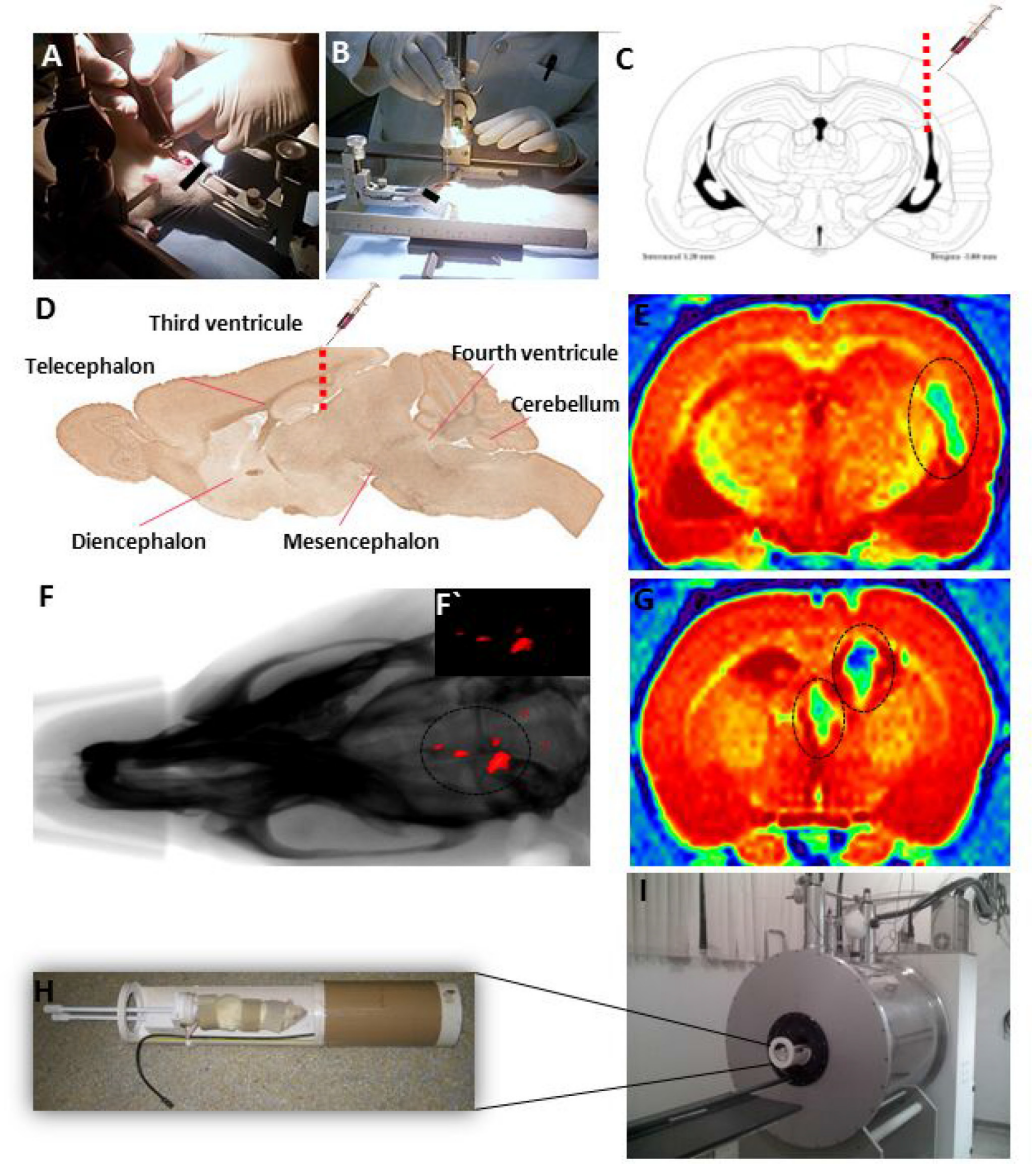
**(A**, **B**) Stereotaxic implantation of EPN cells in brain tumor experimental models. (**C**) Drawing based on Swanson’s Stereotaxic Atlas Guidelines for the indicated coordinates from Bregma -3.80 mm (region of infusion into EPN cells). (**D**) Representative sagittal section demonstrating the injection of MION-Rh labeled EPN cells into the third ventricle. (**F**) Combined fluorescence and X-ray tomography for *in vivo* detection of EPN cells. F´) Fluorescence image detail. (**E**, **G**) MRI monitoring of EPN cells and MION-Rh detection. (**H**) Radiofrequency bobine of animal positioning. I) MRI equipment for small rodent neuroimaging: 2 Tesla superconducting magnet 85310HR.

The progression of tumor growth, generated after stereotaxic implantation in triplicate, of the five EPN cells samples labeled with MION-Rh (Figure 5A, 5B) into the third ventricle (Figure 5C, 5D), was visualized using combined fluorescence and X-ray detection. On day 45, fifteen tumors, from fifteen EPN injected animals, were clearly detectable due to the fluorescently labeled cells (Figure 5F). The success rate of the progression of *in vivo* tumor growth was around 100%.

### MRI monitoring of EPNs growth by MION-Rh detection

**Figure 6:**
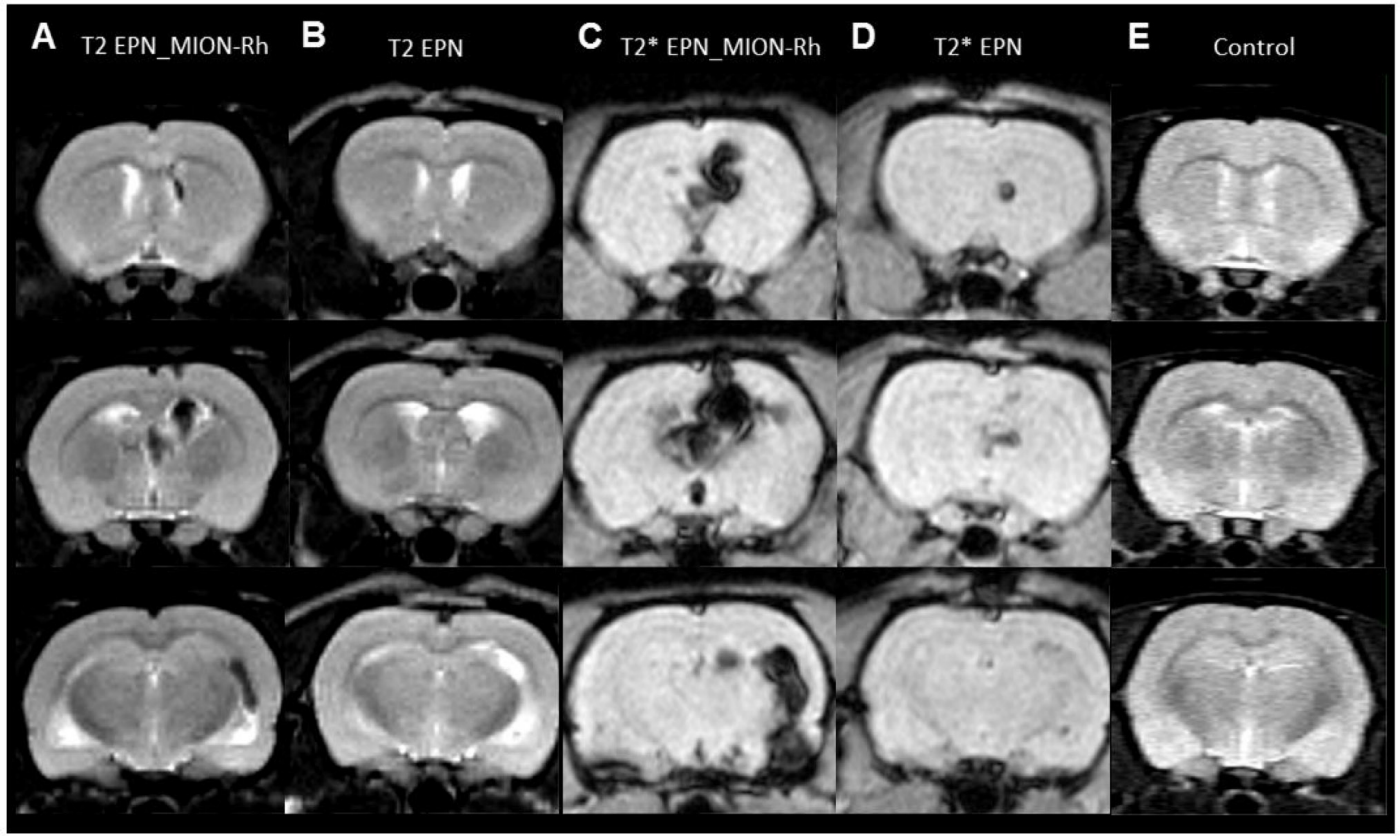
**(A**–**E**) MRI monitoring of *in vivo* EPNs. (A, B, E) T_2_-weighted images. (C, D) T_2_^*^-weighted images. (A, C) Detection of MION-Rh labeled EPN cells. (B, D) Detection of unlabeled EPN cells. (E) Control without any EPN cell transplantation.

Tumor growth was monitored using MRI analysis of MION-Rh labeled EPN cells (Figures 5E, 5G, 6A, 6C); injected unlabeled EPN cells (Figure 6B, 6D); and uninjected controls (Figure 6E). In the animals that were injected with MION-Rh-labeled EPN cells, we observed ‘dark’ hypointense zones in the T_2_-weighted images (Figure 6A), and these zones were more evident in the T_2_^*^-weighted images (Figure 6C). In the animals injected with unlabeled EPN cells, we observed that the T_2_-weighted images (Figure 6B) showed hyperintense zones in the ventricles, whereas the T_2_^*^-weighted images (Figure 6D) displayed hypointense signals in the same area. The control group did not show any signal alteration during MRI (Figure 6E).

### Replication of the histopathological features of the original EPN

**Figure 7:**
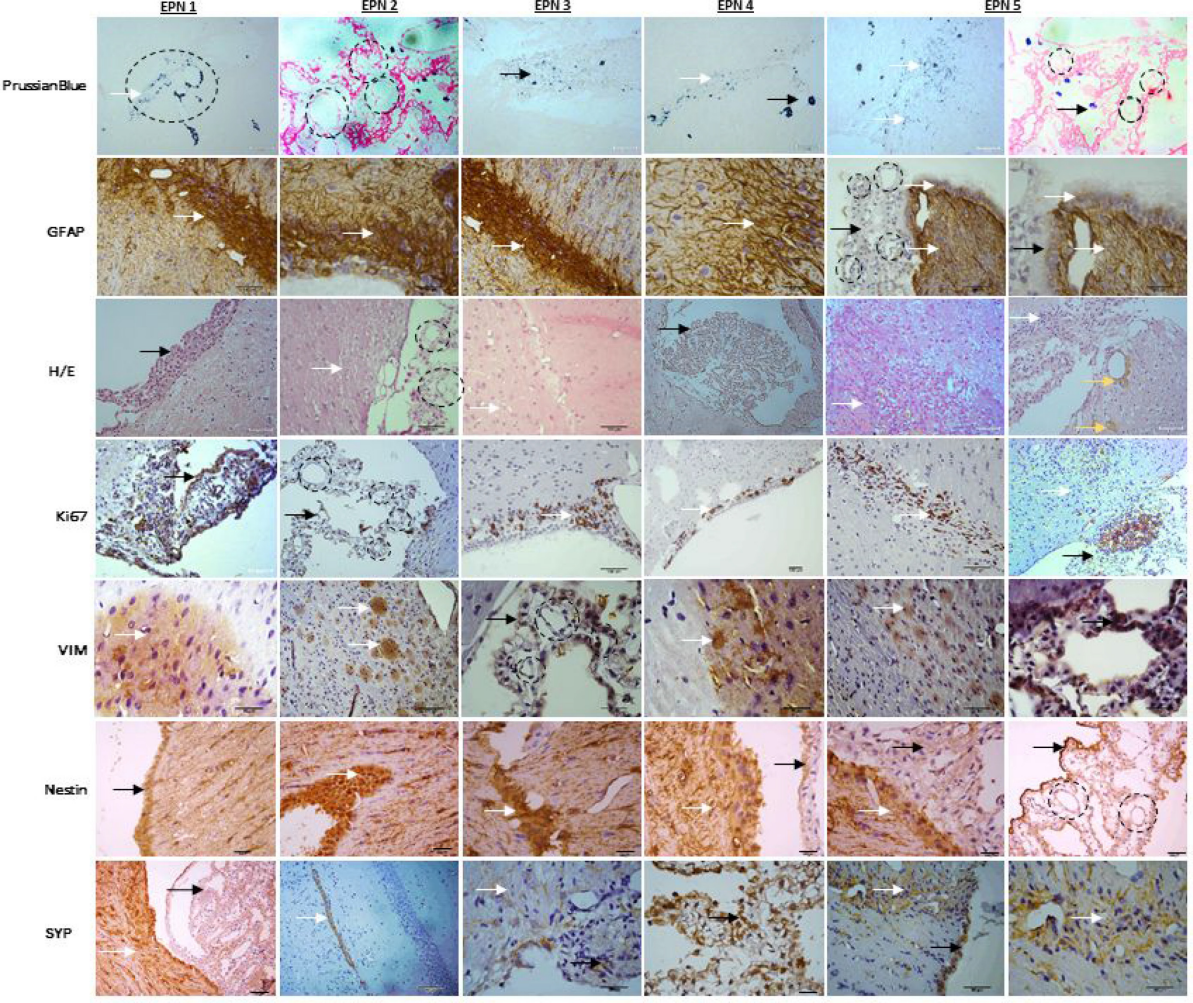
The xenograft tumors displayed characteristic histological features of the original anaplastic EPN, describing the following tests: Immunohistochemical xenograft tumors (EPN1-5) analysis with Prussian Blue for MION-Rh detection, immunohistochemical assay of tumor for glial fibrillary acidic protein (GFAP), hematoxylin and eosin staining, immunohistochemical assay of tumor for Ki67 to detect cell proliferation, vimentin (VIM) to intermediate filament, Nestin to neuron precursor and synaptophysin (SYP) to neuron mature. Black circle: arrangement pseudorosettes with classic intracellular lumens; black arrow: EPN cells that proliferate in the ependymal channel light; white arrow: EPN cells that invade in surrounding region; yellow arrow: hemosiderin granules.

**Figure 8:**
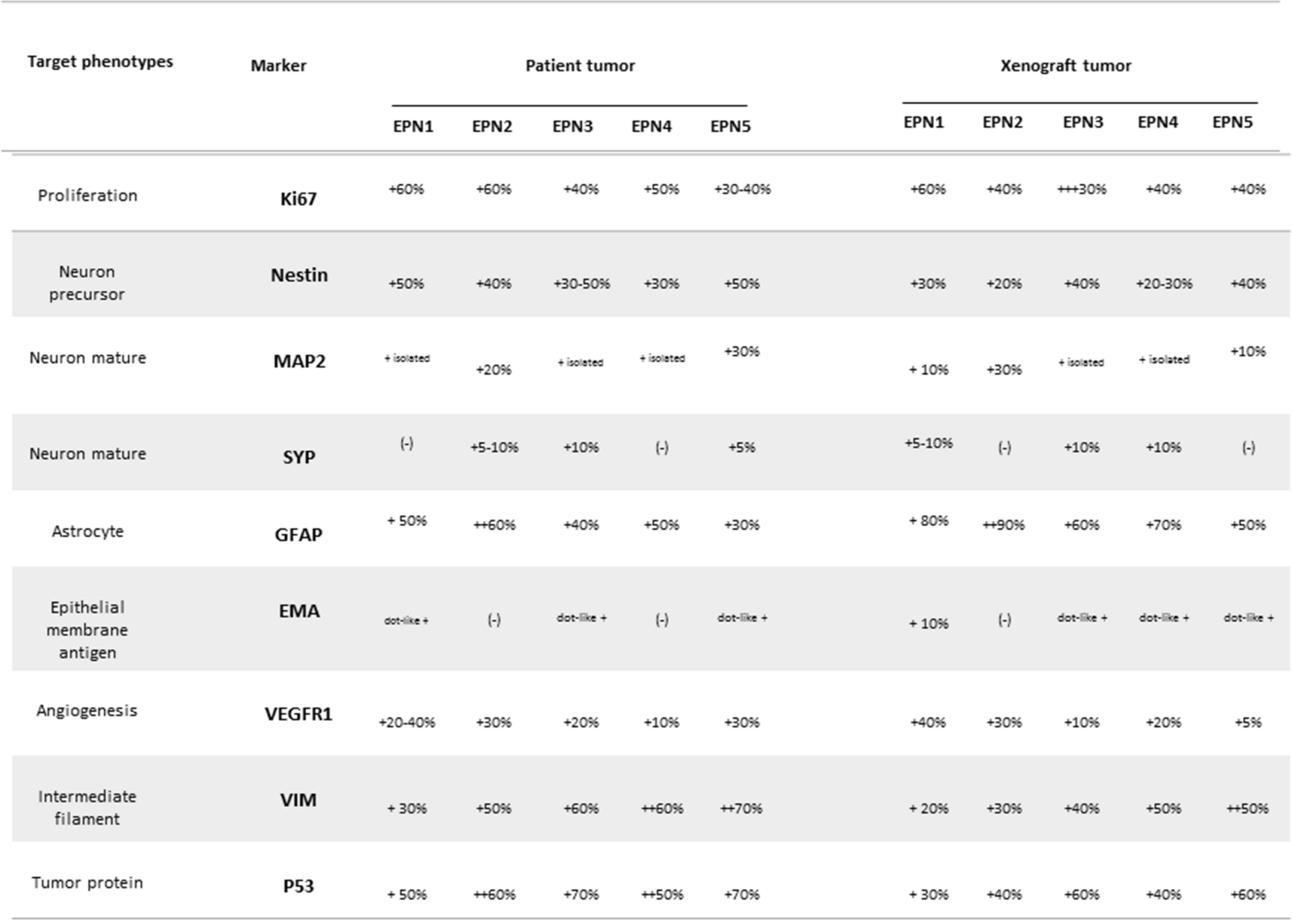
Summary of immunohistochemical features of the patient tumors and of the xenograft tumors during *in vivo* transplantations

To determine whether the xenograft tumors replicated the histopathological phenotypes of the parent tumor (Figure 8), paraffin sections of xenograft tumors (EPN 1-5) were reviewed (Figure 7). The xenograft tumors displayed characteristic histological features of the original anaplastic EPN, including high cellularity, poor differentiation and cells in arrangement pseudorosettes (with classic intracellular lumens), which invaded ependymal channel light and proliferate in surrounding region. This can be visualized by histological analysis for MION-Rh detection using Prussian Blue staining.

Immunohistochemically, the xenograft tumors displayed a profile similar to that of the patient tumor, including a high proliferative (Ki-67) index (30%-60%) homogeneous high-level expression of neuronal precursor marker nestin and tumor protein p53 (Figure 8). The glial marker GFAP was highly expressed in patient and xenograft tumor (Figure 7). Expression of mature neuronal markers MAP2 and SYP were seen in isolated xenograft tumor cells (Figures 7, 8). Nearly, all the xenograft tumor cells stained strongly positive for intermediate neurofilament VIM and KI-67 including tumor cells infiltrating into normal parenchyma (Figure 7). Similar to the patient tumor, staining for epithelial membrane antigen (EMA) was dot-like in the xenograft tumors (Figure 8). The overall expression of VEGF was low in the tumor cells, but strong VEGFR1 positivity was detected in tumor cells forming the perivascular pseudorosettes (Figure 8).

## DISCUSSION

EPNs are relatively rare neoplasms of the CNS that mainly affect children and young adults. Little is known about the aberrant cellular and molecular processes that generate these tumors, and this lack of knowledge has hampered attempts to reduce the high mortality and morbidity rates of the disease. Thus, a disease model is necessary for understanding the biological characteristics of EPNs.

Human EPN cell lines derivation have been successfully established using xenograft tumor tissues [11, 12]. However, the high EPN heterogeneity (such as differences in tumor location, histological grade, clinical behavior, genomic profile) indicates that no single cell culture or preclinical model would entirely recapitulate their complexity. We report herein the establishment of five primary culture directly from seven samples of human pediatric EPNs in the PF. Our straightforward protocol to derive primary EPN cells would be easily usable to investigate EPN biology and explore the potential efficacy of anti-ependymoma drugs *in vitro*.

The obtained cells displayed a fusiform morphology and were arranged in multidirectional bundles in culture. Ultrastructural imaging revealed the presence of large numbers of intermediate glial-like filaments arranged in interwoven bundles. The vast majority of EPN cells contained cytoplasmic extensions, oval nuclei with apparent nucleoli, numerous mitochondria with evident crests; free ribosomes (many of which were present in rosettes); rough endoplasmic reticulum evidenced in cisternae, and slightly dilated Golgi apparatus. The electron microscopic results support the ependymal nature of the neoplastic cells, which is very important for critical diagnosis [15, 16].

The immunophenotypic profile of EPN cells showed that these cells expressed high levels of glial fibrillary acid protein (GFAP), CD44 (a marker involved in cell-cell interactions) and CD90 (a marker of stem/progenitor cells). CD90 was recently identified as a prognostic marker for high-grade gliomas and CD44 as a marker of metastatic potential [17, 18] and coworkers [19] showed that aberrant expression of the cancer stem cell markers CD90 and CD44 contributes to tumoral progression. In addition, CD90 and CD44 expression may explain the tumorigenic potential of these cells because CD44 and CD90 are also known as regulators of migration, invasion and tumor growth. Consequently, these markers may provide targets for molecular therapy in gliomas, including EPNs.

EPN cells also express low levels of CD133 (a glioma stem cell marker), nestin (a marker of immature neural stem cells), CXCR4 (CXC receptor 4, which is involved in tumor migration) and SSEA-3 (stage-specific embryonic antigen 3). Expression of CD133 and SSEA-3 demonstrates the multipotency of the EPN cells, while nestin confirms their neural origin. CXCR4 levels may be useful as a predictive marker of tumor development, growth, metastasis and a therapeutic target [20–22]. Therefore, we propose that GFAP/CD90+/CD44+ EPN cells correlate with the expression of CD133, nestin, CXCR4 and SSEA-3, which could act as markers of enrichment for tumor-initiating cells. In fact, according to our *in vivo* results, the GFAP/CD133+CD90+/CD44+ cells maybe the cell population responsible for EPN dissemination, leading to reduced patient survival due to the inherent chemoresistance of this tumor [17, 18, 23].

The literature describes few details about characterization of distinct immune-phenotyping on EPN. The current study, we focus on studying possible stem cell markers. A barrier to development of immunotherapeutic strategies in pediatric brain tumors is that the immunophenotype of these tumors’ microenvironment has not been defined. Other research needs to be established, since a better understanding of the immunophenotype of EPN would give support to the development of effective immunotherapeutic approaches.

We developed clinically relevant EPN animal models by directly injecting MION-Rh-labeled EPN cells into the third ventricle of immunosuppressed rats. After 45 days, the tumors could be clearly visualized using combined fluorescence and X-ray detection.

MRI analysis proved to be a valuable tool when combined with effective MION-Rh labeling techniques for monitoring the migration trajectory and EPN tumor growth over defined durations. This monitoring was performed by obtaining T2- and T2^*^-weighted MRIs. In animals that were injected with MION-Rh labeled EPN cells, we observed the appearance of ‘dark’ hypointense zones in the T2-weighted images, which were more evident in the T2^*^-weighted images. The presence of MION-Rh changes the magnetic susceptibility in these regions, which accounts for these ‘dark’ zones. In animals that were injected withnon-labeled EPN cells, we observed that the T2-weighted images showed hyperintense zones in the ventricles, i.e., ventricular dilatation, whereas in T2^*^-weighted images, we detected hypointense signals in the same areas. Both of these signal alterations are likely due to the site of the EPN cell injection, which experienced a tissue injury followed by an edema. MRI analyses show potential for monitoring tumor growth of EPN cells efficiently labeled with MION-Rh, which functioned as an outstanding tumor contrast agent.

Following MRI acquisition, the tumor tissue was subjected to histological analysis for MION-Rh detection using the Prussian Blue protocol. Notably, MION-Rh was detected intracellularly in the labeled EPN cells, which indicated that MION-Rh had been retained but not distributed throughout the tumor tissue. This result is likely attributable to iron dilution due to tumor cell proliferation [24].

EPN cells injected into the third ventricle proliferated and migrated to the periventricular region, infiltrating the normal brain parenchyma. Likely, the GFAP/CD133+CD90+/CD44+ EPN cells infiltrated the normal brain parenchyma and have the best chance of escaping surgical resection and of becoming a source of tumor recurrence.

Our xenograft model replicated the histopathological features of the original tumor, demonstrated that this GFAP/CD133+CD90+/CD44+ EPN cells repeated *in vivo* transplantations, thereby maximizing the preservation of biological phenotypes of the original patient tumor, including pseudorosettes, a histological hallmark of EPN. Using human-specific antibodies, we showed that the xenograft tumor cell were all of human origin.

The cell cultures obtained in this work were able to generate tumor compatible with human anatopathological EPN pattern in spite of been grown in media containing serum (10% FBS). Serum is known to cause differentiation of primary glioblastoma stem cells [25] but it did not compromise the tumor generation potential of the EPN primary cells obtained in this work.

Recent identification of cancer stem cells in various human malignancies has provided more insight into tumor initiation, propagation and therapy resistance [26–28]. In EPNs, restricted populations of radial glial cells have been identified as candidate stem cells from various subgroups of EPNs [29, 30]. However, the limited availability of *in vitro* model systems has hampered efforts to understand the biology of these tumors. Successful EPN treatment requires a thorough understanding of the disease’s biological characteristics and of the behavior of its stem cells.

## MATERIALS AND METHODS

Seven samples of human primary anaplastic EPNs (WHO grade III) obtained from the posterior fossa of 1–10-year-old pediatric patients were submitted to resection and analysis at the Pediatric Oncology Institute, Grupo de Apoio ao Adolescente e à Criança com Câncer (GRAACC), Department of Neurology and Neurosurgery, Escola Paulista de Medicina (EPM) - Universidade Federal de São Paulo (UNIFESP), São Paulo, SP, Brazil. All patients granted their informed consent for the study, as determined by the Pediatric Oncology Institute, GRAACC - (UNIFESP- local ethical committee).

The grading of EPNs, according to existing WHO criteria, is still defined by morphological patterns (2016 CNS WHO). Nonetheless, it is expected that continuing studies of the molecular characteristics of EPNs will provide more precise and objective means of subdividing these tumors, allowing for more narrowly defined tumor groups^2^. In this manner, the pathologic anatomy of EPNs were evaluate by stainning of glial fibrillary acidic protein (GFAP) positive, epithelial membrane antigen (EMA) dot-like positive, Ki67 proliferation marker above 40%, p53 positive and above 50%, intermediate filament (VIM) positive, neuron precursor (Nestin) above 30% and angiogenesis (VEGFR1) (Figure 8).

### Establishment of a primary cell culture from EPN samples

Fresh seven EPN samples were washed and minced in phosphate-buffered saline (PBS) (1X), followed by enzymatic dissociation with 0.3% collagenase-I (Sigma-Aldrich). The isolated cells were resuspended in Dulbecco’s Modified Eagle’s Medium-Low Glucose (DMEM-LG, Gibco/Invitrogen Corporation) supplemented with 200 mM L-Glutamine, an antibiotic–antimycotic solution (10,000 U/mL sodium penicillin, 10,000 µg/mL streptomycin sulfate and 25 µg/mL amphotericin B - Gibco/Invitrogen Corporation) and 10% Fetal Bovine Serum (Gibco/Invitrogen Corporation). Cells were subsequently seeded in 25 cm^2^ culture flasks and maintained at 37° C, 5% CO_2_ at fourth passage. The culture medium was changed every other day.

### Immunophenotyping of EPN-initiating cells by flow cytometry

The cell-surface expression of specific markers in primary cell cultures of EPN at fourth passage was analyzed using commercially available human monoclonal antibodies following the manufacturers’ instructions. The cells were stained with the selected monoclonal antibodies and incubated in the dark for 30 minutes at room temperature. For intracellular staining, cells were first fixed with FACS Lysing solution (BD Biosciences, San Jose, CA, USA) and then permeabilized with Permeabilization Solution 2 (BD Biosciences, San Jose, CA, USA). The following human antibodies were used: - GFAP FITC, CD133 PE (clone: 133/2; Milteniy Biotec, Bergisch Gladbach, Germany), Nestin APC, CD44 PE, SSEA-3 FITC, CD90 APC, and CXCR4 PE (all from BD Biosciences, Pharmingen, San Diego, CA, USA) and the related isotype controls or fluorescence minus one (FMO). Data were acquired using a FACSAria flow cytometer (BD Biosciences, San Jose, CA, USA) and analyzed using FACSDiva software (BD Biosciences, San Jose, CA, USA) or FlowJo Software (TreeStar, Ashland, OR, USA).

### Transmission electron microscopy (TEM) of EPNs

The preparation of cultured EPN cells for transmission and scanning electron microscopy was performed using ACLAR^®^ film. EPN cells at fourth passage adhered on ACLAR^®^ film were fixed in 1% glutaraldehyde for two hours at room temperature and then stored at 4° C. Next, the cells were washed in cacodylate buffer (0.1 M, pH 7.2) twice for 15 minutes each and then stored in cacodylate buffer overnight. Post-fixation was performed in 2% osmium tetroxide in cacodylate buffer (0.1 M, pH 7.2) for 1 hour at 4° C, followed by another two 15-minute washes in the same buffer. After dehydration by incubation in ethanol (70% once and 90% twice, 15 minutes each), the cells were embedded in Epon resin diluted in propylene oxide (1:1) and incubated at 48° C with agitation for 24 hours. The cells were then transferred to pure Epon resin at 60° C for 48 hours, until the resin had completely polymerized. Semithin and ultrathin sections were obtained using a Porter Blum ultramicrotome. The ultrathin sections (70 nm) were placed on copper grids and stained with uranyl acetate and lead citrate. The grids were studied and photographed under a TEM (Philips CM100).

### *In vitro* EPN cell labeling with multimodal iron oxide nanoparticles (MION) conjugated to Rhodamine-B (Rh-B) (MION-Rh)

Approximately 10^3^EPN cells at fourth passage were plated into 24-well plates. The cells were incubated overnight (approximately 18 hours) in DMEM-LG with 40 μg Fe/mL MION-Rh at 37° C and 5% CO_2_. After incubation, the culture medium was removed, and the cells were washed twice with PBS (1X) to remove residual extracellular MION-Rh. The labeled cells were treated with 0.25% TrypLE Express (Gibco/Invitrogen Corporation). Cells were immediately harvested, visualized and manually counted using 0.4% Trypan Blue (Gibco/Invitrogen Corporation) under an inverted microscope (IX51 Olympus, Tokyo, Japan).

### Intracellular MION-Rh detection in labeled EPN cells

EPN cells at fourth passage were washed twice with PBS (1X) and fixed with 4% paraformaldehyde. The fixed cells were subsequently subjected to fluorescence analysis using diamidino-2-phenylindole (DAPI, Sigma-Aldrich) to label the cell nuclei and an Rh-B filter (530 nm and 550 nm) to detect the MION-Rh. All cells were analyzed using a fluorescence microscope (IX51 Olympus, Tokyo, Japan).

### Animal ethics statement

All the experimental procedures were performed in accordance with the guidelines for animal experimentation determined by the UNIFESP Care Committee. This protocol was approved by the Committee on the Ethics of Animal Experiments of the UNIFESP. In addition, ethical conditions were maintained, assuming all international rules of animal care outlined by the International Animal Welfare Recommendations and in accordance with local institutional animal welfare guidelines.

### Orthotopic transplantation into complex immune deficiency rats

The animals (male Wistar rats) were treated with immunosuppressant drugs, anesthetized with ketamine (55 mg/kg) and treated with xylazine (11 mg/kg) for implantation of the EPN cells. The hair was then removed from the top of the head. The animal was subsequently fixed to the stereotaxic apparatus (Stoelting^®^, model 51700) using in-ear and upper teeth bars. After making a skin incision on the dorsal region of the skull and removing the periosteum, a trepanation of the bone cap was performed using a dental drill. The implantation position was determined and marked on the bone according to Swanson's Stereotaxic Atlas guidelines at the following coordinates: 0.8 mm anteroposterior, 1.4 mm latero-lateral, and a depth of 3.8 mm. A Hamilton syringe was used to implant 10^4^ EPN cells at third passage in 10 µL of culture medium into the third ventricle. The cells were slowly injected over a 10-minute period. The syringe was kept in position for an additional 2 minutes before being withdrawn. The syringe was slowly raised until it was completely removed from the brain in order to avoid drawing the injected solution back into the needle. The bone was then reassembled using bone wax and the skin sutured using cotton thread. Tumor development was monitored for 45 days. Experiments were in triplicate for each EPN primary cell samples.

### *In vivo* EPN development analysis by molecular imaging

After EPN cell inoculation, tumor development was monitored using an *in vivo* imaging device, Bruker model MSFXPRO. Throughout image acquisition, animals were placed in dorsal recumbency and remained anesthetized with inhaled 2% isoflurane in oxygen at 2 L/min. Initially, the skull images were acquired by X-ray. The fluorescence of the labeled cells was evaluated using the excitation (540 nm) and emission (585 nm) of MION-Rh. The images were acquired using CareStream instrument (MS FX PRO, DT, USA) and evaluated using multiplex location software.

### Magnetic resonance imaging (MRI) EPN analysis

MRI brain scans were obtained in a 2 Tesla/30 cm horizontal superconducting magnet 85310HR (Oxford Instruments, Abingdon, UK) interfaced to a Bruker Avance AVIII console (Bruker-Biospin, Ettlingen, GE) with Paravision 5.1 software (Bruker, Ettlingen, GE) (Figure 5H, 5I). A Crossed Saddle radiofrequency coil [31] was used as a head probe in animals anesthetized with ketamine/xylazine (95/12 mg/kg, i.p.). A T_2_-weighted RARE (Rapid Acquisition with Refocused Echoes) sequence (TR = 5000 ms, TE = 40.5 ms, RARE factor = 8, 4 averages, 6 minutes/animal) was used in a volume of 32 × 32 × 24 mm^3^ covered by a 128 × 128 matrix and 2-mm slice thickness without gaps (12 slices), generating a spatial resolution of 250 × 250 μm^2^. Immediately after RARE acquisition, a T2^*^-weighted image, using a FLASH (Fast Low Angle Shot) sequence (TR = 500 ms, TE = 15 ms, flip angle = 30°, 8 averages, 6 minutes/animal) was acquired. For this image, a volume of 32 × 32 × 24 mm^3^ was covered by a 192 × 192 matrix and 2-mm slice thickness without gaps (12 slices), generating a spatial resolution of 167 × 167 μm^2^. MRI was analyzed qualitatively compared to control animals. In the presence of MION-Rh-labeled EPN cells “dark” regions of hypointensity appear.

### Immunohistochemical (IHC) staining of EPN tissues

After image acquisition, the animals were anesthetized and transcardially perfused with a buffered saline solution and 4% paraformaldehyde (PFA). The brains were removed and stored in PFA for 24 hours and cryoprotected in a 40% sucrose solution for 48 hours. Coronal sections were cut to 40 μm in thickness using a cryostat (Leica) and stained using standard procedures for hematoxylin-eosin and Prussian Blue staining and for IHC staining we used human primary antibodies included the following: antisynaptophysin (anti-SYP) (1:200), glial fibrillary acidic protein (GFAP) (1:200), vimentin (VIM) (1:200), epithelial membrane antigen (EMA) (1:100), MAP-2 (1:200), Ki-67 (1:20), p53 (1:100) (Abcam, Inc.), anti-Nestin (NES) (1:500) and VEGFR-1 (1:150) (Epitomics, Inc.). After slides were incubated with primary antibodies, the appropriate biotinylated secondary antibodies were applied, and the final signal was developed using the 3,30-diaminobenzidine substrate kit for peroxidase. A negative control was performed by replacing the primary antibody with 1% BSA in PBS. The IHC staining was assessed by combining the intensity, scored as negative (–), low (+), medium (++), and strongly positive (+++) and extent of immunopositivity expressed as the percentage of positive cells.

## CONCLUSIONS

We successfully established and characterized *in vitro* and *in vivo* EPN models for studying the molecular pathways necessary for EPN growth and progression. Our study showed that direct orthotropic implantation of a fresh surgical specimen led to the development of a clinically relevant animal model of pediatric EPN, a tumor for which no permanent cell lines are available. We demonstrated that GFAP/CD133+CD90+/CD44+ EPN cells maintained key histopathological and growth characteristics of the original patient tumor.
